# Dip effect on the orientation of rock failure plane under combined compression–shear loading

**DOI:** 10.1038/s41598-024-53497-z

**Published:** 2024-02-22

**Authors:** Lijun Sun, Pengcheng Li, Binyu Luo, Xiaoyun Liu, Tengda Huang, Yuan Su

**Affiliations:** 1 State Key Laboratory of Safety and Health for Metal Mines, Maanshan, 243000 Anhui China; 2 National Engineering Research Center for Efficient Recycling of Metallic Mineral Resources, Maanshan, 243000 Anhui China; 3 School of Resource and Environmental Engineering, Wuhan University of Science and Technology, Wuhan, 430081 Hubei China; 4 Hubei Key Laboratory for Efficient Utilization and Agglomeration of Met Allergic Mineral Resource, Wuhan, 430081 Hubei China

**Keywords:** Engineering, Civil engineering

## Abstract

Shear failure often occurs in engineering rock mass (such as inclined pillar) in gently inclined strata. Prediction and characterization the orientation of shear failure plane is the foundation of rock mass engineering reinforcement. In this paper, sandstone samples are used to perform uniaxial and shear tests to obtain the basic mechanical parameters. Then, by employing the numerical method, the combined compression–shear loading tests were carried out for inclined specimens varied from 0° to 25° at an interval of 5°, to obtain the dip effect on the orientation of rock failure plane. The results show that the failure plane of rock changes with the change of dip angle of rock sample. Based on the Mohr–Coulomb criterion, the ultimate stress state of rock was characterized under combined compression–shear loading. The ultimate strength of rock is equal to the ratio of the stress circle radius of rock under combined compression–shear condition to the stress circle radius of rock under uniaxial compression condition, multiplied by the uniaxial compressive strength. The fracture angle of rock was defined under combined compression–shear loading. A theoretical model was developed for predicting the fracture angle. The developed model could be characterized by internal friction angle, dip angle of rock sample and Poisson's ratio. Finally, the numerical results of the fracture angle were analyzed, which are consistent with the predicted results of the model. The investigation shows that the rock fracture angle has a dip effect, which decreases with the increase of the inclination angle of the sample. The research results provide a new means to identify the potential failure plane of engineering rock mass, and lay a theoretical foundation for calculating the orientation of rock fracture plane.

## Introduction

**Figure 1:**
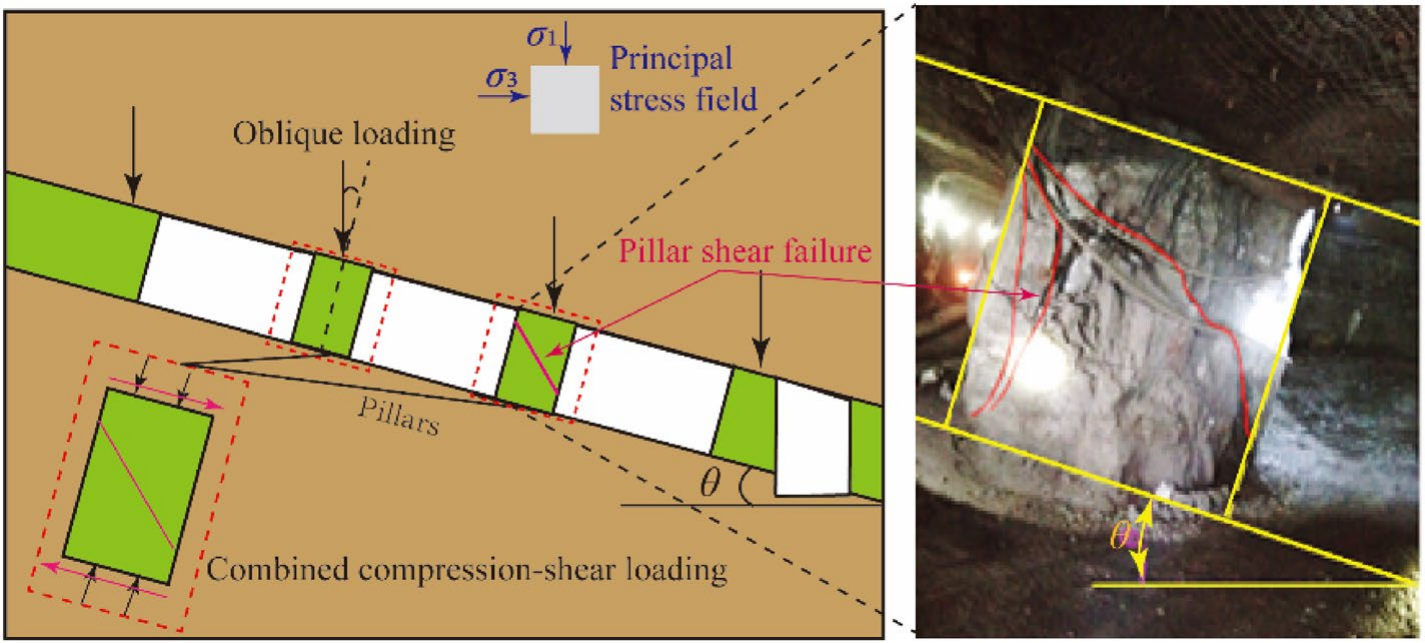
Pillar under the combined action of compression and shear.

With the development of social economy, more and more mineral resources are consumed. these mineral resources are exploited in underground, often using methods such as room and pillars, and caving, longwall mining methods^1,2^. In underground mining, the most critical thing is to ensure the stability of underground rock mass structures such as stopes and tunnels. The stability of these structures is affected by mining methods, faults, hydrogeology, rock formation inclination and other factors^3^. Once the structures become unstable, it may cause surface subsidence. Mine pillars are an important part of the underground space structure^4^. Ensuring the stability of the mine pillars is a prerequisite for safe mining^5^. Once the pillar is unstable, it may induce the collapse of the goaf and lead to the surface subsidence^6^. In the process of mining underground deposits, influenced by the dip angle of the ore seam, the underground space structure is subjected to the combined action of compression and shear load. Compression and shear loads changed the loading path^7–9^ of rock mass engineering, and induced shear failure^10–12^. For example, for the inclined pillar, which reduced its bearing capacity, showing the dip effect on bearing capacity^13–16^, increasing the risk of instability of pillar and seriously affecting the safe mining^17^, as shown in Fig. 1. Therefore, it is very important to study the strength of rock under the combined compression–shear loads to deeply understand the strength characteristics of rock structure and its safety evaluation.

To carry out the combined compression–shear loading test of rock, which is similar to the loading path of rock mass engineering in the laboratory, is the premise of understanding the deformation and failure of rock mass engineering. At present, there are three ways to realize the combined compression–shear loading of rock, and its principle is to transform the uniaxial compression load on the universal test machine (MTS) into the combination of compression–shear load. So as to realize the combined compression–shear loading test of rock. The first way is to grind the two ends of the rock sample into an inclined plane^18,19^to realize the compression–shear loading. The second way is to add a pair of single slope devices to the compression testing machine to realize the combined compression–shear loading^20–22^. The third way is to add a pair of devices with double slopes to the universal testing machine to realize the combined compression–shear loading^23–25^. The three methods mentioned above have their own advantages and disadvantages.

Based on the first combined compression–shear loading approach of rock, Xu et al.^18,19,26^ adopted the methods of test and numerical simulation to research the dynamic and static mechanical behavior and failure mode of inclined cylindrical rock. The results show that the rock is shear-dominated failure. Xu et al.^27^ studied that the failure mode of inclined specimen is affected by micro-damage and global stress field accumulated during preloading. The study shows that the shear fracture mode of rock under dynamic load or high preload changes to the axial splitting mode near the surface of specimen under low preloading.

Based on the combined compression–shear loading approach of the second kind of rock, He et al.^20,21,28^ studied the mechanical behavior and strength properties of basalt, granite and coal samples under combined compression–shear loading. The results show that the decrease of rock strength and elastic modulus with the increase of specimen inclination, which is closely related to the strength and modulus under uniaxial compression. With the increase of specimen inclination, the failure mode of the specimen gradually changed from axial separated fracture to shear failure. Jessu et al.^22^ carried out compression–shear loading tests on gypsum samples and sandstone under the background of inclined pillar, and studied the compression–shear strength of gypsum and sandstone, which decreased with the increase of rock dip angle. Wu et al.^29^ combined with acoustic emission technology to study the effects of different dip angle on the mechanical properties and fracture behavior of coal samples. With the increase of inclination angle, the crack initiation threshold (CI) and crack damage threshold (CD) of coal samples increased at first and then decreased. The failure mode of coal samples changes from tension crack failure (0°-5°) and tension-shear combined failure (10°) to simple shear failure (15°–25°). Cao et al.^30^ carried out compression–shear loading tests on granite samples. The results show that the fracture behavior of rock depends to a large extent on the mechanism of shear stress component. The larger the dip angle is, the larger the shear component is, the more significant the shear failure of rock is. Chen et al.^31,32^ carried out compression–shear loading tests on granite at high temperature and yellow sandstone after low temperature freezing and thawing, and studied the mechanical behavior of rock under temperature conditions and dip angle. The results show the degree of shear fracture is higher under higher temperature treatment, and the influence of the inclination angle on the failure mode of saturated yellow sandstone is more significant than that of the freeze–thaw effect.

Based on the third rock compression–shear combined loading approach, Xu et al.^24,30^ carried out dynamic and static combined compression–shear tests on granite. The results show that the deformation and strength have obvious compression–shear coupling effect (that is, loading path sensitivity). It is shown that the larger the sample angle is, the smaller the failure strength is. Zhou et al.^33^ studied the dynamic characteristics of granite under combined compression–shear loading. A high-speed camera was used to collect images of artificial speckle samples. The transverse tensile strain field and the maximum shear strain field are calculated and given by digital image correlation (DIC) method. Affected by the dip angle, the evolution of rock strain amplitude density obeys bimodal (bimodal) Weibull distribution. The first peak (first peak 1) and the second peak (second peak 2) are dominated by the local deformation evolution of the matrix part and the failure part of the specimen, respectively. Luo et al.^25^ carried out compression–shear combined loading tests of rock-like materials. The results show that the shear failure occurs and the bearing capacity decreases with the increase of the inclination of the sample. Then, according to the shear failure mode and strength characteristics of the tested rock, based on Mohr–Coulomb shear failure theory, the rock strength model under the combined compression–shear loads was established, and the effective characterization of rock strength dip effect is realized.

Based on the summary of the above research results, it is found that the mechanical behavior, failure mode and strength characteristics of rock under combined compression–shear load are dependent on the loading path. The main results are as follows: with the increase of loading angle (the dip angle of sample), the rock is dominated by shear failure, and the strength decreases gradually. What is more important is that the compression–shear strength is always less than its uniaxial compressive strength. These results lay a foundation for the investigation of compression–shear failure criterion and orientation of rock failure plane. However, at this stage, it is mainly focused on object to explore the mechanical behavior, failure mode and strength characteristics of rock under compression–shear loading for different loading paths in the laboratory. Among them, the failure form of rock specimen is the most intuitive expression and important feature of rock failure mechanism. How to effectively characterize or predict the orientation of failure plane is an important means to explain rock failure mechanism. It also provides a theoretical basis for the reinforcement of weak surface or potential broken section of rock mass engineering^34^. Based on this, the dip effect on the orientation of rock failure plane is studied by numerical simulation and theoretical methods. The fracture angle is used to characterize the orientation of rock failure plane, the correlation characteristics between rock fracture angle and sample dip angle are analyzed. The mapping relationship between sample dip angle and rock fracture angle is established, and the theoretical model of the orientation of rock failure plane is developed. The purpose of the research is to provide a theoretical basis for accurately judging the orientation of rock failure plane, and to provide reference for rock mass engineering reinforcement orientation.

## Numerical simulation of dip effect on rock failure plane

### Model parameters calibration

**Figure 2:**
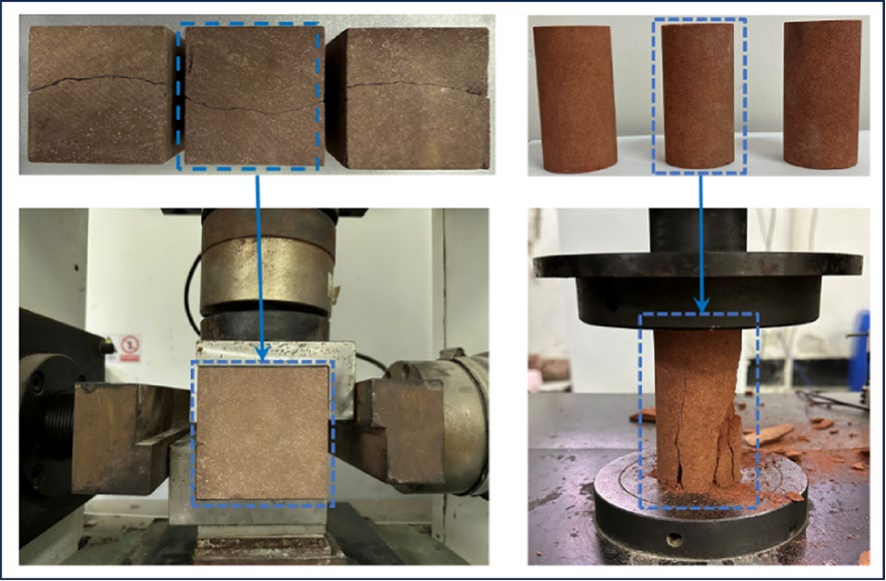
The shear and uniaxial compression tests.

**Figure 3:**
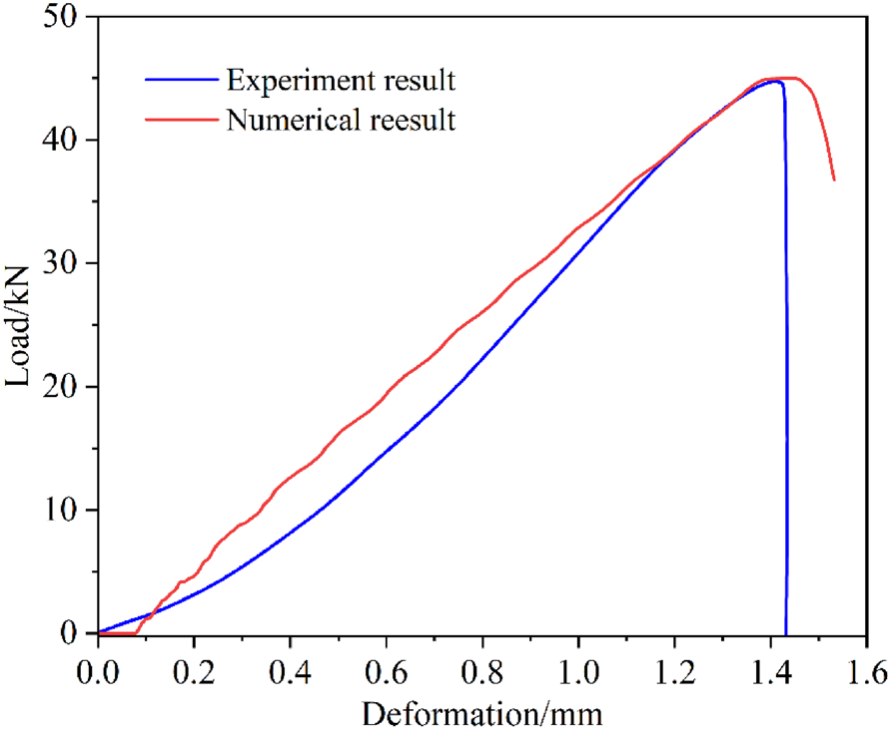
Comparison diagram of parameter verification.

Under the premise of inputting enough accurate parameters, numerical simulation is an important means to study the mechanical behavior of rock. To obtain reliable numerical model parameters, the uniaxial compression tests and shear experiments of red sandstone were carried out as shown in Fig. 2. According to the deformation-load curves and shear strength obtained from the test, the numerical model is assigned as Mohr–Coulomb constitutive model, and the model parameters are adjusted to match the curve of the laboratory experiments, as shown in Fig. 3. The matched mechanical parameters are shown in Table 1. The mechanical parameters at this time are used as the basis to simulate the inclined loading test (combined compression–shear loading test) under different inclination angles.

**Table 1:** Mechanical parameters employed in numerical model.

Density (kg/m^3^)	Bulk (GPa)	Shear (GPa)	Friction (°)	Cohesion (MPa)	Tension (MPa)	Poisson's ratio
2600	6.02	5.14	31.8	4.93	3.8	0.2

### Numerical simulation process

**Figure 4:**
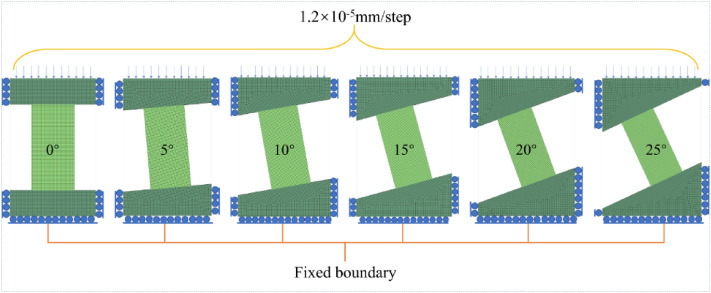
Numerical model with the different dip angle.

The numerical model is a plane-stress model, composed of an upper base, rock sample, and lower base. The rock sample width is 50 mm, the height is 100 mm, and the mesh size is 1 mm. The model bottom and the sides of the model top are fixed. The model top is applied a fixed velocity boundary with 1.2 × 10^-5^ mm/step, until the post-peak stage. The upper base and the lower base are assigned as an elastic constitutive model, and the rock sample is assigned as Mohr–Coulomb constitutive model. The sample dip was varied from 0° to 25° at 5° increments, as shown in Fig. 4.

### Numerical results

**Figure. 5:**
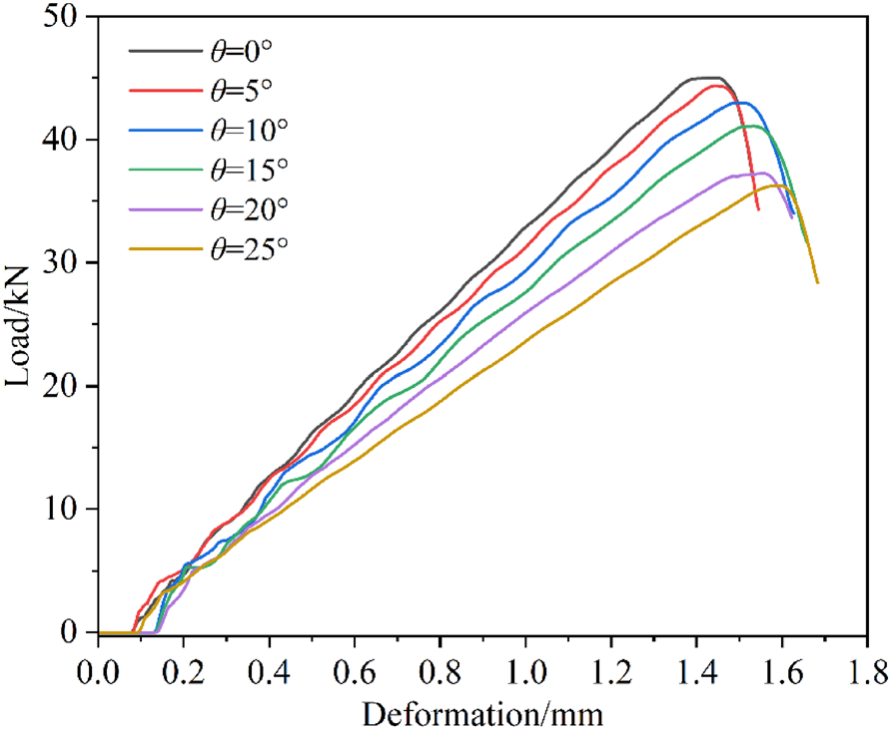
Stress–strain curves of rock samples with different inclination angles.

Figure 5 shows the load-deformation curve under different loading inclinations (samples with different inclination angles). It can be seen from the diagram that the maximum bearing capacity of the rock sample decreases with the increase in inclination, which is consistent with the phenomenon observed by other scholars^16,20^. At the same time, as the inclination increases, the deformation required to reach the peak load gradually increases, indicating the elastic modulus decreases with the increase of dip angle^16^.

**Figure 6:**
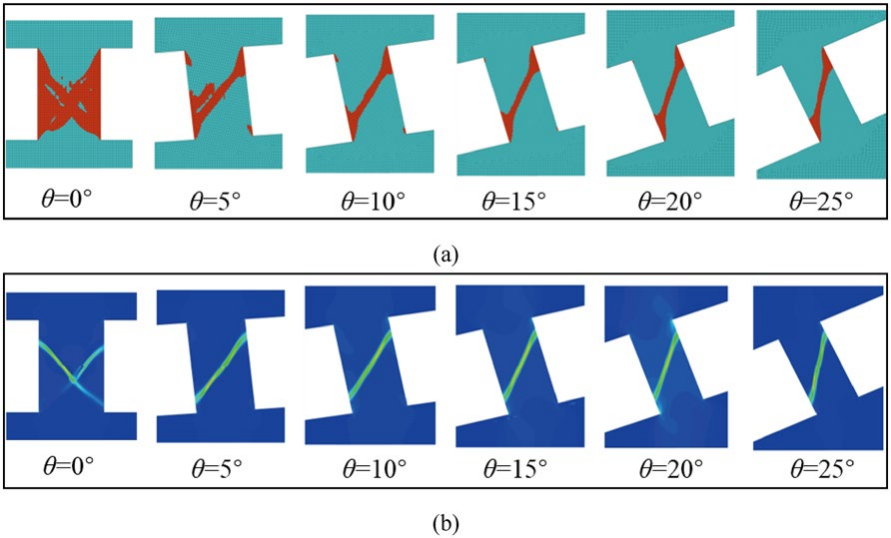
Dip effect on rock shear failure plane; (**a**) Dip effect of plastic zone evolution; (**b**) dip effect of shear band.

Figure 6 shows the evolution of the plastic zone and the shear band with the inclination angle. From the Fig. 6, it can be found that the evolution law of the plastic zones is consistent with that of the nonlocal deformation zone, and their positions also coincide with each other. In the process of uniaxial loading, the normal stress and shear stress of the unit at different positions are different, and the parts with tremendous stress (the parts with tremendous shear stress) yield first. At 0° inclination angle, the stress is symmetrical from left to right, so conjugate shear failure occurs. When there is an inclination angle, the stress on the rock block is no longer symmetrical, and the shear stress on the unit at a certain angle is often more significant, so a single shear failure will eventually occur. Once a block yields, it cannot keep pace with the deformation of other elastic blocks, and a band with prominent color is formed, that is, the local deformation band. With continuous loading, the bearing capacity of the block in the plastic zone is further weakened, the plastic deformation is further increased. When it reaches a certain threshold, macroscopic fracture occurs. Thus, it can be seen that with the increase of the inclination angle, the single slope evolution appears in the plastic zone, indicating that the specimen has one-sided shear failure, which is consistent failure law with the test results^20,21,25^.

## Theoretical model for the angle of rock potential failure plane

### Characterization of ultimate stress state by Mohr–Coulomb criterion under combined compression–shear loads

In the numerical process, the end faces of the specimen are subjected to the combined action of compression and shear loads. It is necessary to consider the failure criterion of both compression load and shear load. Among the failure criteria proposed by predecessors, Mohr–Coulomb criterion satisfies these conditions well, and it is also one of the most widely used in rock engineering and geotechnical engineering.

Generally speaking, the study of rock failure criterion based on Mohr–Coulomb strength theory is divided into three steps: ① in the Mohr stress space, the stress circle characterizing the stress state is derived under the combined compression–shear loads. ② Find the Mohr Coulomb strength envelope equation. ③ the strength criterion of rock is established by combining the stress circle equation with the strength envelope equation.

Now, according to the three steps mentioned above, the failure criterion of rock is derived under the combined action of compression and shear. Under the combined compression–shear loading, the relationship between compressive stress *σ* and shear stress *τ* on any inclined plane in the sample is as follows:

1 $$\left( {\sigma - \frac{{\sigma_{\theta } }}{2}} \right)^{2} + \left( {\tau - \frac{{\tau_{\theta } }}{2}} \right)^{2} = \left( {\frac{{\sigma_{\theta } }}{2}} \right)^{2} + \left( {\frac{{\tau_{\theta } }}{2}} \right)^{2}$$

Equation (1) could be expressed as a circle on the *σ–τ* coordinate plane through the origin of the coordinates, the center of the circle is (*σ*_*θ*_/2, *τ*_*θ*_/2) and the radius is $0.5\sqrt {\sigma_{\theta }^{2} + \tau_{\theta }^{2} }$. Compared to traditional stress circles, the center of this stress circle is not on the stress axis.

**Figure 7:**
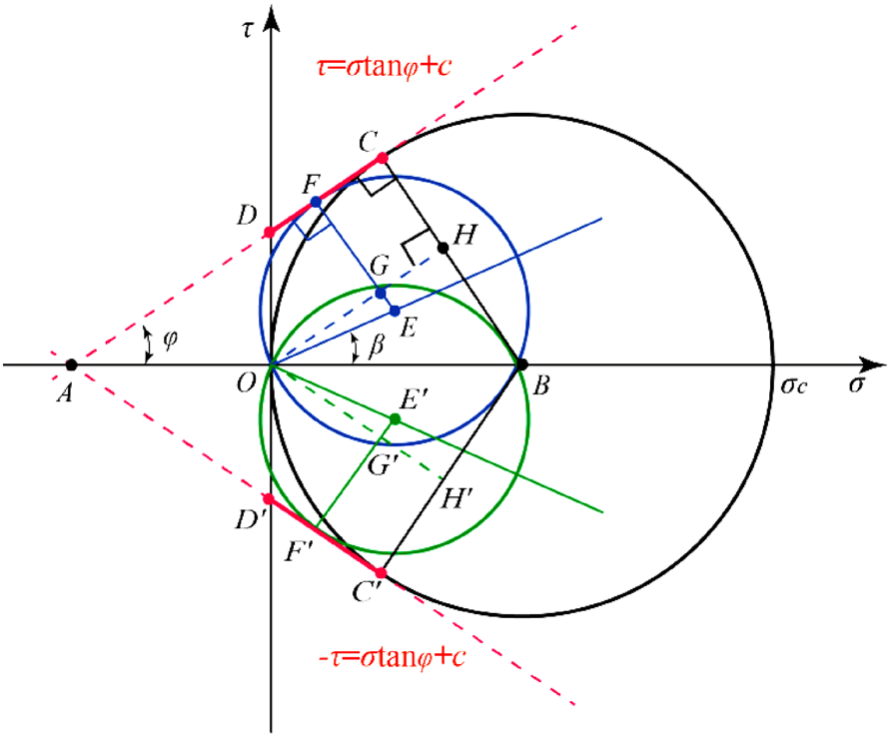
The relationship between stress circle and Mohr–Coulomb strength envelope.

According to Mohr–Coulomb’s theory, the relationship between ultimate stress circle and Mohr–Coulomb envelope of rock under combined compression–shear load, and between ultimate stress circle and Mohr–Coulomb envelope of rock under uniaxial compression is shown in Fig. 7. According to the geometric relationship, the ultimate stress circle radius of rock under uniaxial compression is *OB* = *BC* = 0.5*σ*_*c*_, while the ultimate stress circle radius of rock under combined compression and shear load is *OE*^25^, then

2 $$OE = EF = EG + GF = EG + BC - BH$$

where

3 $$\left\{ \begin{gathered} EG = OE\sin \left( {\varphi - \beta } \right) \hfill \\ BH = OB\sin \varphi = \frac{{\sigma_{c} }}{2}\sin \varphi \hfill \\ \end{gathered} \right.$$

The combination of Eqs. (2) and (3),

4 $$OE = \frac{{\sigma_{c} }}{2}\frac{1 - \sin \varphi }{{1 - \sin \left( {\varphi - \beta } \right)}}$$

where the radius *OE* of the limit stress circle could also be expressed by the compression stress and shear stress component:

5 $$OE = \frac{1}{2}\left[ {\sqrt {\sigma_{\theta }^{2} + \tau_{\theta }^{2} } } \right]$$

Combining Eqs. (4) and (5):

6 $$\frac{1}{2}\left[ {\sqrt {\sigma_{\theta }^{2} + \tau_{\theta }^{2} } } \right] = \frac{{\sigma_{c} }}{2}\frac{1 - \sin \varphi }{{1 - \sin \left( {\varphi - \beta } \right)}}$$

Equation (6) is the limit equilibrium equation of rock stress state under the combined compression–shear load, and the left side of the equation is the actual load of rock. When the left side of Eq. (6) is smaller than the right side, the rock will not fail, otherwise, the rock will fail. In Eq. (6), *β* is the dip of a straight line on which the center of the generalized Mohr’s circle is located. Here, tan*β* is determined by the ratio of shear stress component to compressive stress component at the end face of rock samples:

7 $$\tan \beta = \frac{{\tau_{\theta } }}{{\sigma_{\theta } }} = \frac{\tan \theta }{{2\left( {1 + \nu } \right)}}$$

Combining Eqs. (6) and (7), then

8 $$\frac{1}{2}\left[ {\sqrt {\sigma_{\theta }^{2} + \tau_{\theta }^{2} } } \right] = \frac{{\sigma_{c} }}{2}\frac{1 - \sin \varphi }{{1 - \sin \left( {\varphi - \arctan \left[ {\frac{\tan \theta }{{2\left( {1 + \nu } \right)}}} \right]} \right)}}$$

It can be seen from Eq. (8), when the rock sample is in the limit equilibrium state, if *r*_*θ*_ is used to represent the stress circle radius of the rock under the combined compression–shear load, *R*_*c*_ represents the stress circle radius of the rock under uniaxial compression, then λ is the ratio of the stress circle radius of the two loading states.

9 $$\lambda = \frac{{r_{\theta } }}{{R_{c} }} = \frac{OE}{{BC}} = \frac{1 - \sin \varphi }{{1 - \sin \left( {\varphi - \arctan \left[ {\frac{\tan \theta }{{2\left( {1 + \nu } \right)}}} \right]} \right)}}$$

The comparative Eqs. (8) and (9) could be obtained that the strength *σ*_*θc*_ of rock under the combined compression–shear load is expressed as:

10 $$\sigma_{\theta c} = \frac{{r_{\theta } }}{{R_{\theta } }}\sigma_{c} = \lambda \sigma_{c}$$

Obviously, under the Mohr–Coulomb criterion, the compression–shear strength of rock is equal to the ratio of the stress circle radius of rock under uniaxial compression to that of rock under combined compression–shear loading multiplied by the uniaxial compressive strength of rock. It could also be expressed by uniaxial compressive strength *σ*_*c*_, internal friction angle *φ*, Poisson's ratio *v* and rock sample inclination angle *θ*.

### Characterization of dip effect on rock failure plane

**Figure 8:**
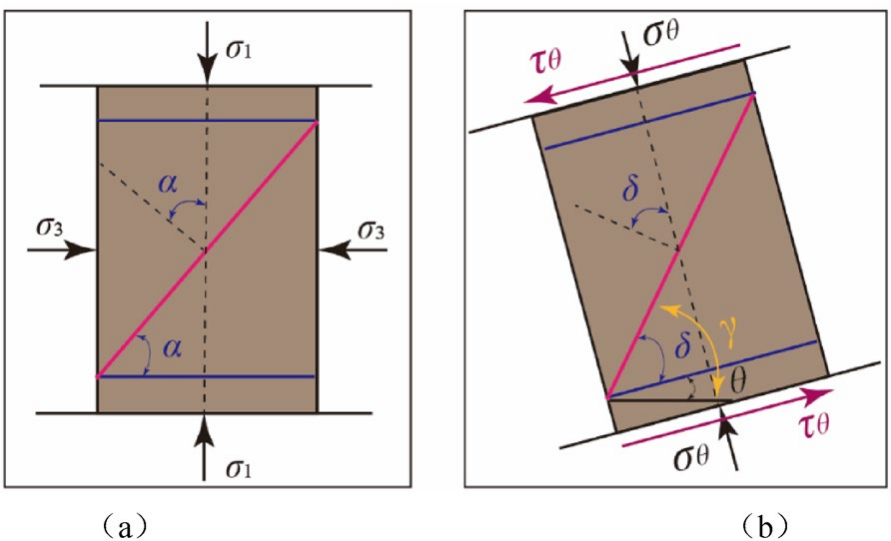
The concept for the angle of the rock failure plane; (**a**) triaxial compression loads; (**b**) combined compression–shear loads.

Under the condition of Mohr–Coulomb criterion, the angle *α* of failure plane is named rock fracture angle, which the normal vector of failure plane makes an angle with the direction of maximum principal stress under uniaxial or triaxial compression, as shown in Fig. 8a. Similarly, under combined compression–shear load, the angle *δ* of rock fracture by which the normal vector of failure plane makes an angle with the direction of compression stress, as shown Fig. 8b. It is said that the angle between the normal vector of failure plane and the horizontal plane is the dip angle of the failure plane, which is represented by the symbol *γ*. It should be noted that in uniaxial or triaxial compression, the fracture angle is equal to the dip angle of the failure plane.

**Figure 9:**
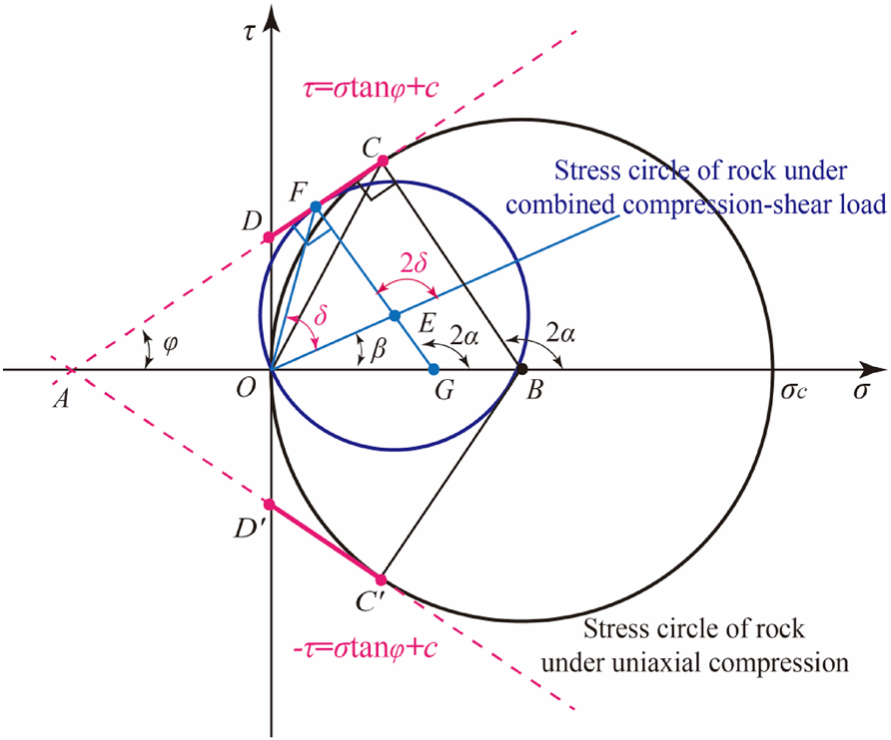
The angle of failure plane in Mohr’s stress space under combined compression–shear loading.

The fracture angle is generally characterized by the angle of internal friction. The relationship between rock fracture angle *α* and internal friction angle *φ* under uniaxial or triaxial compression is shown in Fig. 9.

11 $$2\alpha = \frac{\pi }{2} + \varphi$$

Similarly, the relationship between the fracture angle *δ* of rock under the combined compression–shear load and the fracture angle *α* of rock under compression is shown in Fig. 9. According to the geometric relationship in Fig. 9.

12 $$2\alpha = 2\delta + \beta$$

Combining Eqs. (11) and (12), the fracture angle *δ* is obtained under combined compression–shear loading.

13 $$\delta = \alpha - \frac{\beta }{2} = \left( {\frac{\pi }{4} + \frac{\varphi }{2}} \right) - \frac{\beta }{2}$$

In Eq. (7), the fracture angle *δ* of rock is expressed by internal friction angle *φ*, Poisson's ratio *ν*, and rock sample inclination angle *θ*, as follows

14 $$\delta = \left( {\frac{\pi }{4} + \frac{\varphi }{2}} \right) - \frac{1}{2}\arctan \left[ {\frac{\tan \theta }{{2\left( {1 + \nu } \right)}}} \right]$$

According to Mohr–Coulomb criterion, the fracture angle of rock is fixed under uniaxial or triaxial compression. From the Eq. (14), the fracture angle of rock under the combined compression–shear condition is smaller than that of rock under compression condition. Under combined compression–shear condition, the fracture angle of rock is different, and it changes with the change of dip angle of rock sample. The Eq. (14) could be used to predict the fracture angle of rock samples at different dip angles.

In addition, according to the definition of rock failure plane angle, it is obtained that the dip angle of the rock *γ* is equal to the sum of the fracture angle *δ* and the sample inclination angle *θ*, as shown in Fig. 8, that is:

15 $$\gamma = \delta + \theta = \left( {\frac{\pi }{4} + \frac{\varphi }{2}} \right) - \frac{1}{2}\arctan \left[ {\frac{\tan \theta }{{2\left( {1 + \nu } \right)}}} \right] + \theta$$

Thus, it can be seen that the orientation of the failure plane of the rock can be expressed by the rock fracture angle or the dip angle of the failure plane. Therefore, when strengthening the engineering rock mass, the support scheme can be designed according to the orientation of the potential weak plane of the rock (A plane that may fail).

## Discussion

### The relationship among parameters *θ*, *ν* and *β*

When rock is under combined compression–shear loading, the ratio of shear stress to compressive stress on the end face is expressed by tan*β*. The ratio is also the slope of the straight line at the center of the generalized stress circle. The dip angle *β* has an important influence on the compression–shear strength and the fracture angle of the rock, which reflects the proportional relationship of the compression and shear stress component, and its magnitude is determined by the sample inclination angle *θ* and the Poisson's ratio *v* of the rock.

**Figure 10:**
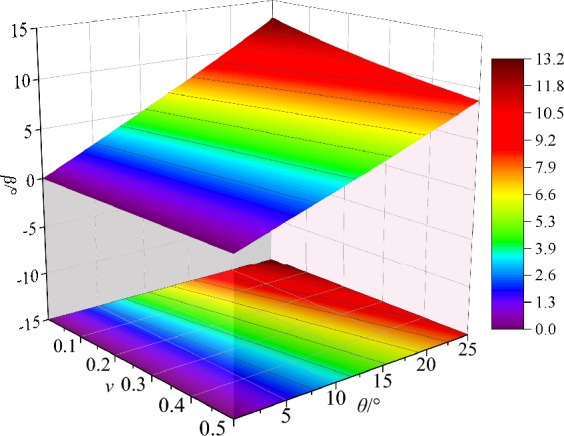
The relationship between *β* and *θ* and Poisson's ratio ν.

If Poisson's ratio is equal to *ν* ∈ [0, 0.5] and rock sample inclination *θ* ∈ [0°, 25°]. The value range of inclination angle *β* is calculated by using Eq. (7), as shown in Fig. 10. When Poisson's ratio is constant, the inclination angle *β* increases with the increase of sample inclination *θ*, and the larger the Poisson ratio is, the greater the inclination angle *β* is. When the inclination angle *θ* of the sample is constant, the inclination angle *β* decreases gradually with the increase of Poisson's ratio, and the greater the decrease degree of the inclination angle *β* is under the larger sample inclination angle. According to the relationship between the inclination angle *β* and the sample inclination angle *θ* with Poisson's ratio *ν*, the effects of sample inclination angle *θ* and Poisson's ratio *ν* on the compression–shear strength and fracture angle *δ* are further analyzed.

### Dip effect on failure plane

**Figure 11:**
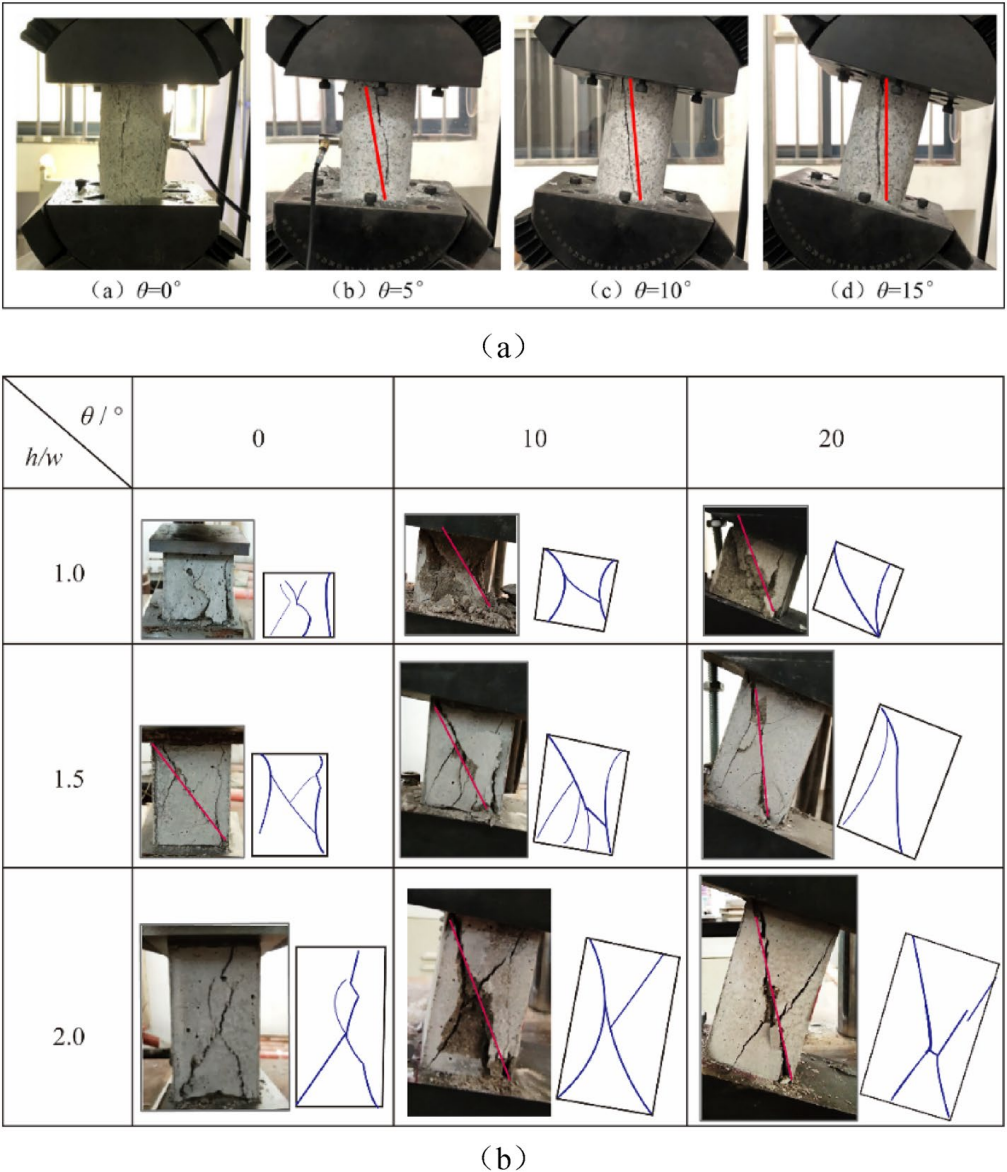
Failure plane of rock under combined compression–shear test; (**a**) dip effect on the angle of failure plane for marble rock^21^; (**b**) dip effect on the angle of failure plane for rock-like material^25^.

The test results have shown, under combined compression–shear loading (except for uniaxial compression), the failure mode of rock is a single inclined plane, as shown in Fig. 11^20,21,25^. It can be seen from the Fig. 11 that the shear failure plane of the rock is closely related to the inclination of the rock sample, and it varies with the change of the inclination of the sample. Under uniaxial compression, the failure mode of rock or fracture features of brittle is affected by strain rate and lithology^35,36^, such as splitting, shear, cone and so on. Under the combined loading of rock-like materials and coal samples, with the increase of dip angle, the orientation of failure plane changes gradually, and the failure plane shows a strong dip effect.

**Figure12:**
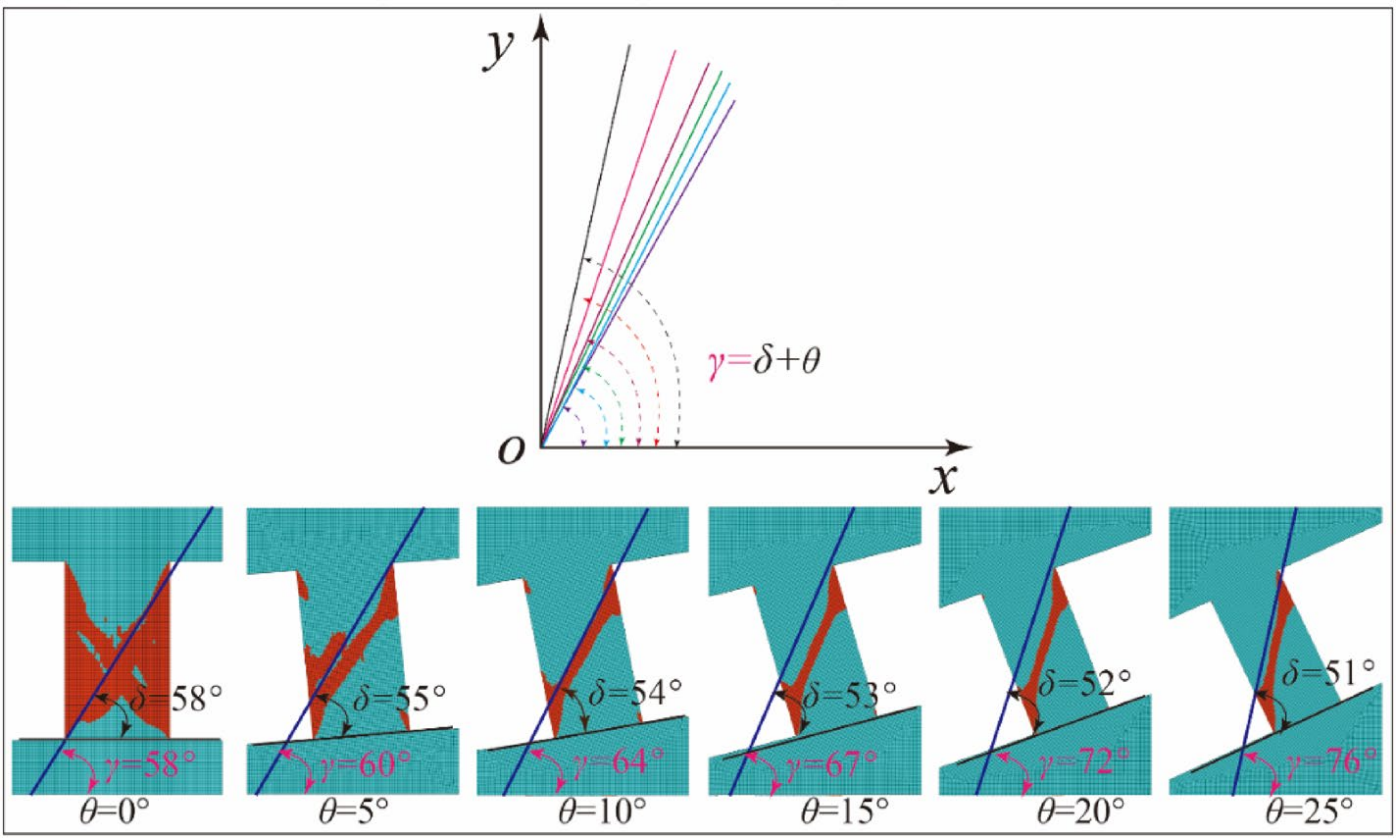
The angle of the rock failure plane and the inclination angle between the failure plane and the horizontal plane.

Similarly, in the compression–shear numerical loading test of rock, the plastic zone of rock develops in an inclined narrow zone, as shown in Figs. 6 and 12, which shows that the failure of the sample is also a single oblique shear failure, which is consistent with the experimental results. In order to determine the fracture angle and dip angle of failure plane, the cloud map of plastic zone obtained by numerical simulation is imported into AutoCAD software, and the angle between inclined plastic zone and sample end face and between inclined plastic zone and horizontal plane is measured, as shown in Fig. 12. That is, the fracture angle and dip angle of rock failure plane are obtained under different sample dip angles. The statistics of the fracture angle and dip angle of failure plane and the dip angle of the sample are shown in Table 2. With the dip angle of the sample, the fracture angle decreases gradually, while the dip angle of failure plane increases.

**Table 2:** The relationship between the inclination of the sample and the orientation of the failure plane.

*θ* (**°**)	*δ* (**°**)	*γ* (**°**)
0	58	58
5	55	60
10	54	64
15	53	67
20	52	72
25	51	76

### Comparison of results for the angle of failure plane: numerical method vs. theoretical model

**Figure 13:**
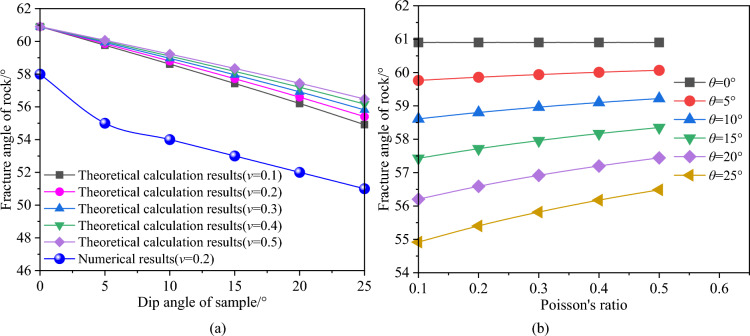
Relationship between angle of failure plane and inclination angle and Poisson's ratio; (**a**) given Poisson's ratio; (**b**) given the inclination angle of rock sample.

Figure 13 shows the effect of sample inclination and Poisson's ratio on shear fracture angle. When Poisson's ratio is constant, the fracture angle of rock decreases with the increase of sample inclination angle, and the fracture angle reaches the maximum when the dip angle is equal to 0°. And the larger the Poisson's ratio, the smaller the decrease of the fracture angle, as shown in Fig. 13a. When the inclination angle (except 0°) of the sample is constant, the fracture angle increases with the increase of Poisson's ratio, and the larger the dip angle of the sample, the greater the decrease of the fracture angle, as shown in Fig. 13b. This shows that the fracture angle of rock is larger, and the rock is easier to fail under the condition of larger dip angle and smaller Poisson's ratio.

**Figure 14:**
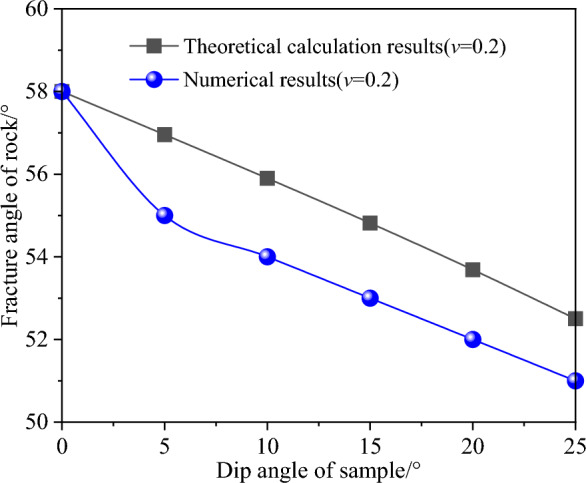
Comparison of calculation results for the angle of failure plane: numerical method vs. theoretical model.

In Fig. 13, when Poisson's ratio is in the range of 0 to 0.5, the fracture angle predicted by theoretical model is always larger than that of numerical simulation (Poisson's ratio is equal to 0.2). This is because the failure angle under uniaxial compression is calculated by using the Mohr–Coulomb criterion. On this basis, the fracture angle of rock under other dip angles is predicted. It can be seen that the calculation of the failure angle of rock is very important under uniaxial compression. In view of this, under uniaxial compression, based on the fracture angle calculated by numerical method (Instead of the fracture angle is not 45° plus half of the internal friction angle), the new fracture angles of rock under different dip angle of sample are predicted by the theoretical model. And compared with the numerical results, show that the predicted results are in good agreement with the numerical results, as shown in Fig. 14.

## Conclusions

1. The orientation of the rock failure plane has a dip effect under the combined compression–shear loading. Both rock fracture angle and dip angle of the failure plane were defined, they change with the varying of dip angle.
2. A theoretical model for predicting rock fracture angle was developed from Mohr–Coulomb criterion, which can be characterized by internal friction angle, dip angle of rock sample and Poisson's ratio. This developed model is an extension of the Mohr criterion for calculating the rock fracture angle.
3. The simulation results of rock fracture angle are in good agreement with the theoretical prediction results. With the increase of the inclination angle of the sample, the fracture angle decreases, while the dip angle of the failure plane increases. This study provides a theoretical basis for the reinforcement of potential failure plane of engineering rock mass.

## Data Availability

All data generated or analysed during this study are included in this published article.
